# Motion Artifacts (MA) At-Rest in Measured Arterial Pulse Signals: Time-Varying Amplitude in Each Harmonic and Non-Flat Harmonic-MA-Coupled Baseline

**DOI:** 10.3390/bios15090578

**Published:** 2025-09-04

**Authors:** MD Mahfuzur Rahman, Mamun Hasan, Zhili Hao

**Affiliations:** Department of Mechanical and Aerospace Engineering, Old Dominion University, Norfolk, VA 23529, USA; mrahm009@odu.edu (M.M.R.); mhasa004@odu.edu (M.H.)

**Keywords:** motion artifacts (MA), arterial pulse measurement, baseline drift, harmonics, time-varying system parameters (TVSP), microfluidic-based tactile sensor, dynamic systems, time-frequency analysis (TFA), tissue-contact-sensor (TCS) stack

## Abstract

Motion artifacts (MA) cause great variability in a measured arterial pulse signal, and treatment of MA solely as a baseline drift (BD) fails to eliminate its effect on the measured signal. This paper presents a study on the effect of MA at rest (<0.7 Hz) on measured arterial pulse signals using a microfluidic-based tactile sensor. By taking full account of the dynamic behavior of the transmission path from the true pulse signal in an artery to a measured pulse signal at the sensor, the tissue-contact-sensor (TCS) stack, an analytical model of MA in a measured pulse signal is developed. In this model, the TCS stack is treated as a 1DOF system for its dynamic behavior; MA is quantified as the displacement (i.e., BD) and time-varying system parameters (TVSP) of the TCS stack. The mathematical expression of MA in a measured pulse signal reveals that while BD remains as low-frequency additive noise, TVSP causes time-varying harmonics in a measured pulse signal. Further time-frequency analysis (TFA) of measured pulse signals validates the existence of TVSP and, for the first time, reveals its effect on a measured pulse signal: time-varying amplitude in each harmonic and non-flat harmonic-MA-coupled baseline.

## 1. Introduction

Numerous clinical studies based on medical instruments (i.e., tonometry and ultrasound) have revealed significant physiological and pathological values of arterial pulse signals [1,2,3,4]. Owing to their low cost and ease to use, various sensors based on micro/nano-fabrication technology have been explored as an alternative to medical instruments for arterial pulse measurement [5,6,7,8]. Although photoplethysmography (PPG) sensors have been utilized for pulse measurement for several decades [3], it is mainly used at the index finger [3] and the wrist [9,10] and is unsuitable for pulse measurement at the carotid artery (CA). Due to its proximity to the aorta, the pulse signal at the CA is more indicative of the cardiovascular (CV) system [1,2]. In this regard, tactile sensors have been developed for pulse measurement at the CA [6]. As compared to other micro/nano-fabricated tactile sensors, microfluidic-based tactile sensors offer several advantages, such as great fabrication simplicity, low electrical noise, and easy adjustment in sensor design [6,11]. Yet, like medical instruments and PPG sensors, measured pulse signals using tactile sensors suffer from measurement variability [6,12].

Regardless of design variations, in essence, a tactile sensor is composed of a deformable microstructure and a transducer underneath, sitting on a substrate [12]. In pulse measurement, a tactile sensor is manually held and pressed against an artery with contact pressure *P_c_* to establish tissue–sensor contact. Then, the true pulse signal in the artery can go through the transmission path: overlying tissue, tissue–sensor contact, and sensor, namely the tissue-contact-sensor (TCS) stack, and deforms the microstructure and registers as an electrical signal (i.e., a measured pulse signal) by the transducer. Although the working principle for pulse measurement is straightforward, experimental studies have identified all the factors in the TCS stack: overlying tissue (i.e., individuals), *P_c_*, the sensor used, and sensor alignment, that cause non-negligible variability in measured pulse signals [5,12].

To make things worse, a measured pulse signal is subject to motion artifacts (MA), even when an individual under measurement is at-rest, due to body motion, respiration, and finger jittering [3,9,10,12]. Given the unavoidable nature of MA, various signal-processing algorithms have been developed to estimate and subtract MA from a measured pulse signal to obtain a pulse signal free of MA [13,14,15], which is commonly referred to as the measured arterial pulse waveform (APW). Various features of measured APW and its time derivatives are then extracted as arterial indices to quantify CV conditions [3,12,13]. As compared with a pulse signal (>1 Hz), MA at rest represents low-frequency (<0.7 Hz) signals [9]. Since MA alters the distance of the sensor relative to the artery, intuitively, MA is directly equated with the baseline drift (BD) in a measured pulse signal and is deemed as low-frequency additive noise [9,10,13,14,15]. As such, all the existing signal-processing algorithms for MA at rest, including wavelet-based filtering and Empirical Mode decomposition (EMD), are built upon the low-frequency nature of BD [13,14,15]. These filtering and decomposing techniques aim to separate low-frequency components as BD from high-frequency components in a measured pulse signal. After this stage, the baseline in a filtered pulse signal is usually non-flat, and cubic spline estimation (CSE) is then brought in to estimate a curve for the filtered pulse signal as the extra BD to force it with a flat baseline [14,15]. This pulse signal with a flat baseline is ultimately treated as measured APW. Yet, the true pulse signal in an artery itself is a collection of harmonics of the heart rate [4,16]. As a low-frequency signal, this extra BD adds distortion to the obtained APW. Since filtering and EMD introduce their own distortion to a measured pulse signal, different algorithms applied to the same measured pulse signal yield different estimations of BD and result in different measured APWs [15].

This study is built upon a 2DOF (degree-of-freedom) model for interpreting the variability in measured pulse signals, where the TCS stack is treated as a 1DOF model to fully account for its dynamic behavior involved in pulse measurement [12]. In other words, the TCS stack is not rigid and does not simply undergo a displacement shift to transmit the true pulse signal in an artery from its one end to its other end, the sensor. Instead, the TCS stack undergoes time-varying deformation in response to the true pulse signal, and then its dynamic behavior is involved in pulse measurement. It is well-established that prestress in a solid alters its mechanical properties and consequently its system parameters when treated as a dynamic system [17]. Overlying tissue is analogous to a solid, and thus the system parameters of the TCS stack are expected to be altered by BD. As such, MA needs to be quantified as (1) BD and (2) time-varying system parameters (TVSP) of the TCS stack. As compared to BD, TVSP rides on each harmonic of the true pulse signal and thus smears each harmonic in a measured pulse signal [12].

Due to MA, a measured pulse signal is nonstationary, and its time-frequency analysis (TFA) becomes necessary to examine the effect of MA on it. Currently, the TFA of a nonstationary signal via various techniques, such as continuous wavelet transform (CWT), varying-frequency complex demodulation (VFCDM), and Hilbert vibration decomposition (HVD), is a hot research topic in many fields [18,19,20,21]. TFA has been applied to PPG signals for respiration rate extraction [19,20]; TFA of a measured pulse signal for the effect of MA at rest on a measured pulse signal has not been reported yet, to the best knowledge of the authors.

This study aims to examine the effect of MA at rest on a measured pulse signal via time-frequency analysis. In particular, this study focuses on validating the existence of TVSP and examining its effect on a measured pulse signal, since the existence of TVSP in the TCS stack is neglected in all the literature on MA. First, an analytical model of MA in a measured pulse signal is developed, where the TCS stack and the artery are treated as a 2DOF model, while the system parameters of the TCS stack are determined by overlying tissue, the sensor, and *P_c_*. MA is added to the TCS stack as its displacement (BD) and TVSP. MA is mathematically related to a measured pulse signal (i.e., a calculated pulse signal at the TCS stack). The related numerical calculation is conducted in MATLAB (2024a) to obtain a calculated pulse signal with pre-defined MA. Due to its simplicity, the HVD method is employed to conduct the TFA of a measured pulse signal. A calculated pulse signal with pre-defined MA is used to validate the HVD method. Measured pulse signals using a microfluidic-based tactile sensor are then analyzed using the HVD method. For the first time, this study reveals that MA causes time-varying amplitudes in each harmonic and non-flat harmonic-MA-coupled baseline in a measured pulse signal. As compared to all the existing studies on MA at rest, the novelty of this study lies in: (1) a full account of the dynamic behavior of the TCS stack in pulse measurement instead of BD only (i.e., treating the TCS stack as rigid), and (2) the TFA of a measured pulse signal for a clear manifestation of MA in it.

## 2. Materials and Methods

### 2.1. Arterial Pulse Measurment Using a Microfluidic-Based Tactile Sensor

**Figure 1:**
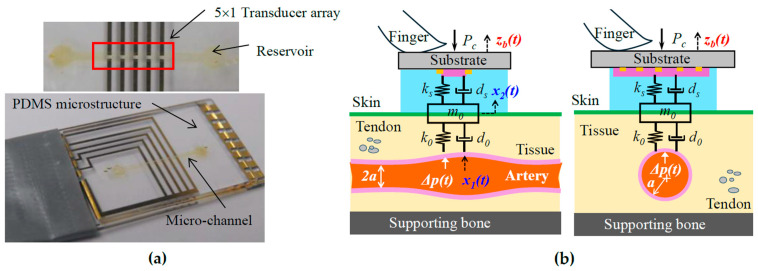
(**a**) Pictures of a microfluidic-based tactile sensor; (**b**) schematics of arterial pulse measurement using the sensor.

Figure 1a shows a microfluidic-based tactile sensor for pulse measurement. The sensor contains a polydimethylsiloxane (PDMS) microstructure embedded with a microchannel underneath, sitting on a Pyrex substrate with five pairs of metal electrodes. After filling the microchannel with electrolytes via a reservoir, both reservoirs are sealed with PDMS. Electrolytes in the microchannel and five pairs of metal electrodes aligned along the channel length form a 5 × 1 resistive transducer array. A pulse signal deforms the microstructure, which translates to resistance changes in the transducers.

As shown in Figure 1b, to acquire a pulse signal, the sensor is flipped over and is manually held and pressed against the CA with *P_c_*. The transducer array, with a length of 6mm, is aligned perpendicularly to the artery length so that at least one transducer can be easily aligned above the CA, with a radius of 3 mm, for acquiring a clear pulse signal. The true pulse signal, pulsatile pressure *Δp*(*t*), in the CA goes through the TCS stack: overlying tissue, tissue–sensor contact, and the sensor, and is recorded as a measured pulse signal *x*_2_(*t*) by the sensor. Accompanying the *Δp*(*t*) is the arterial wall displacement *x*_1_(*t*). The details about the sensor design, fabrication, associated electronics, and LabVIEW programs for its operation and real-time monitoring of a measured pulse signal can be found in [22,23].

Arterial pulse measurements at the CA using the sensor were approved by the Institutional Review Board (IRB) at Old Dominion University. The details about the measurement protocol can be found in [11]. Since this study focuses on examining the effect of MA, specifically TVSP, on a measured pulse signal, we analyze the measured pulse signals from only one healthy 32 yr-old male subject at rest under two CV conditions: pre-exercise and 5 min post-exercise.

### 2.2. An Analytical Model of MA in a Measured Pulse Signal

For simplicity, the TCS stack is assumed to behave linearly, and the transduction in the sensor is neglected so that a measured pulse signal is the displacement output. As shown in Figure 1b, upon *P_c_*, tissue–sensor contact is established and the TCS stack is formed. Due to its deformability, the tissue forms a 1DOF system, and the microstructure in the sensor forms another 1DOF system. Tissue–sensor contact joins the mass of the tissue and the microstructure together. As such, the TCS stack forms a 1DOF system with a spring and a damper in parallel on both sides of its mass *m*_0_. While the tissue forms spring *k*_0_ and damper *d*_0_, the microstructure forms spring *k_s_* and damper *d_s_*. The mass *m*_0_ includes contributions from the tissue and the sensor. Note that *P_c_* presets the normal values of *m*_0_, *k*_0_, and *d*_0_ in the TCS stack and a static displacement in the sensor. This static displacement is excluded here, since its effect on a measured pulse signal is accounted for by the preset nominal values. MA causes a time-varying displacement *z_b_*(*t*) at the sensor substrate:

(1) $$z_{b}(t)=z_{b0}e^{j{(\omega }_{b}t+\varphi_{b})}$$

where *z*_*b*0_, *φ_b_*, and *ω_b_* are the amplitude, phase, and angular frequency of *z_b_*(*t*), respectively.

**Figure 2:**
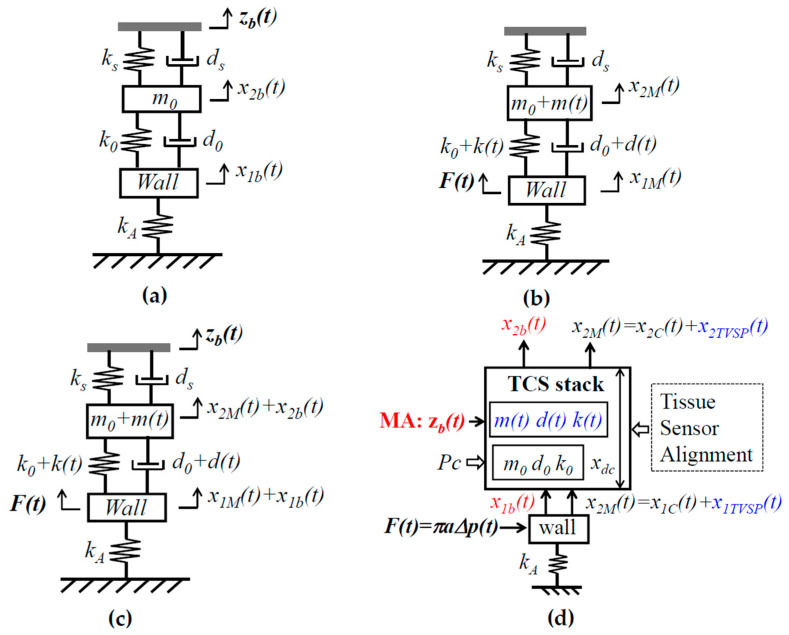
An equivalent 2DOF model of the TCS stack and an artery in pulse measurement. (**a**) Input *z_b_*(*t*) on the sensor substrate; (**b**) input *F*(*t*) on the arterial wall and TVSP arising from *z_b_*(*t*); (**c**) two inputs: *z_b_*(*t*) and *F*(*t*); and (**d**) all the factors in pulse measurement and parameters for their quantification: low-frequency parameters related to MA in red font and TVSP-related parameters in blue font.

As shown in Figure 2a, since the inertia and damping terms of the arterial wall are negligible, relative to its elastic term, the arterial wall is modeled as a spring with stiffness *k_A_*. While one end of the spring is fixed, its other end is connected to the TCS stack. Adding the arterial wall to the TCS stack forms a 2DOF system. The displacement *z_b_*(*t*) at the sensor substrate serves as the base excitation for the 2DOF system and leads to the displacements *x*_1*b*_(*t*) and *x*_2*b*_(*t*) at the arterial wall and the TCS stack, respectively:

(2a) $$\begin{array}{l} {-k_{0}x}_{1b}\left(t\right)-d_{0}\frac{{dx}_{1b}\left(t\right)}{dt}+m_{0}\frac{{d^{2}x}_{2b}\left(t\right)}{{dt}^{2}}+{{(k}_{0}+k_{s})x}_{2b}\left(t\right)+{(d}_{0}+d_{s})\frac{{dx}_{2b}\left(t\right)}{dt}\\ \;\;\;\;\;\;\;\;\;\;\;\;\;\;\;\;\;\;\;\;\;\;\;\;\;=k_{s}z_{b}\left(t\right)+d_{s}\frac{dz_{b}\left(t\right)}{dt} \end{array}$$

(2b) $$(k_{A}+k_{0})x_{1b}\left(t\right)-k_{0}x_{2b}\left(t\right)+d_{0}\left[\frac{{dx}_{1b}\left(t\right)}{dt}-\frac{{dx}_{2b}\left(t\right)}{dt}\right]=0$$

The sensor measures the relative distance between its substrate and the mass. Thus, the measured BD by the sensor is *x*_2*b*_(*t*) − *z_b_*(*t*). The length change in the TCS stack, *x*_2*b*_(*t*) − *x*_1*b*_(*t*), causes the time-varying system parameter (TVSP) of the TCS stack:

(3) $$\begin{array}{c} m=m_{0}+m(t),\;d=d_{0}+d(t),\;k=k_{0}+k(t)\\ \mathrm{with}\;m\left(t\right),\;k(t),\;d(t)\propto x_{2b}(t)-x_{1b}(t) \end{array}$$

where *m*(*t*), *d*(*t*), and *k*(*t*) are TVSP of the TCS stack, whose frequency is <0.7 Hz.

Pulsatile pressure *Δp*(*t*) is considered as the true pulse signal and translates to a force *F*(*t*) acting on the arterial wall [12]:

(4) $$\left(t\right)=F_{0}e^{j{(\omega }_{p}t+\varphi_{p})}=\pi a\Delta p\left(t\right)\;\;\mathrm{with}\;\;\Delta p\left(t\right)=\Delta p_{0}e^{j{(\omega }_{p}t+\varphi_{p})}$$

where *F*_0_, *φ_p_*, and *ω_p_* are the amplitude, phase, and angular frequency of *F*(*t*), respectively.

As shown in Figure 2b, in response to *F*(*t*), the displacements at the TCS stack and the wall are governed by the following:

(5a) $$\begin{array}{c} {-\left(k_{0}+k\left(t\right)\right)x}_{1M}\left(t\right)-\left(d_{0}+d\left(t\right)\right)\frac{{dx}_{1M}\left(t\right)}{dt}+\left(m_{0}+m\left(t\right)\right)\frac{{d^{2}x}_{2M}\left(t\right)}{{dt}^{2}}+\\ {{(k}_{0}+k(t)+k_{s})x}_{2M}\left(t\right)+{(d}_{0}+d(t)+d_{s})\frac{{dx}_{2M}\left(t\right)}{dt}=0 \end{array}$$

(5b) $$\begin{array}{c} \left(k_{A}+k_{0}+k\left(t\right)\right){\cdot x}_{1M}\left(t\right)-\left(k_{0}+k\left(t\right)\right)\cdot x_{2M}\left(t\right)+(d_{0}+d\left(t\right))\\ \cdot \left[\frac{{dx}_{1M}\left(t\right)}{dt}-\frac{{dx}_{2M}\left(t\right)}{dt}\right]=F\left(t\right) \end{array}$$

The solution to *x*_1*M*_(*t*) and *x*_2*M*_(*t*) takes the forms

(6a) $$x_{1M}\left(t\right)=x_{1T}{(t)e}^{j\varphi_{1T}(t)}=G_{1}(m,c,\;k;t)F_{0}e^{j{(\omega }_{p}t+\varphi_{p})}\;\;\mathrm{with}\;\omega_{1T}(t)=\frac{{d\varphi }_{1T}(t)}{dt}$$

(6b) $$x_{2M}\left(t\right)=x_{2T}{(t)e}^{j\varphi_{2T}(t)}=G_{2}(m,c,\;k;t)F_{0}e^{j{(\omega }_{p}t+\varphi_{p})}\;\;\mathrm{with}\;\omega_{2T}(t)=\frac{{d\varphi }_{T2}(t)}{dt}$$

where *x*_1*T*_, *φ*_1*T*_, and *ω*_1*T*_ are the instant amplitude, phase, and frequency of *x*_1*M*_(*t*), respectively; *x*_2*T*_, *φ*_2*T*_, and *ω*_2*T*_ are the instant amplitude, phase, and frequency of *x*_2*M*_(*t*), respectively. The measured pulse signal using the sensor and the wall displacement become

(7a) $$x_{tactile-M}\left(t\right)=x_{2M}\left(t\right)+x_{2b}\left(t\right)-z_{b}\left(t\right)$$

(7b) $$x_{wall-M}\left(t\right)=x_{1M}\left(t\right)+x_{1b}\left(t\right)$$

When free of MA, based on (5), the displacement *x*_2*C*_(*t*) at the TCS stack and the displacement *x*_1*C*_(*t*) at the arterial wall are

(8a) $$\begin{array}{l} x_{1C}\left(t\right)=x_{10}e^{j{(\omega }_{p}t+\varphi_{p}+\varphi_{10})}=G_{10}F_{0}e^{j{(\omega }_{p}t+\varphi_{p}+\varphi_{10})}\\ \mathrm{with}\;G_{10}e^{j\varphi_{10}}=\frac{1}{k_{A}-\frac{\left(d_{0}\omega_{p}j+k_{0})(m_{0}\omega_{p}^{2}-d_{s}\omega_{p}j-k_{s}\right)}{-m_{0}\omega_{p}^{2}+\left[d_{0}+d_{s}\right]\omega_{p}j+\left[k_{0}+k_{s}\right]}} \end{array}$$

(8b) $$\begin{array}{l} x_{2C}\left(t\right)=x_{20}e^{j{(\omega }_{p}t+\varphi_{p}+\varphi_{20})}=G_{20}F_{0}e^{j{(\omega }_{p}t+\varphi_{p}+\varphi_{20})}\\ \mathrm{with}\;G_{20}e^{j\varphi_{20}}=\frac{1}{k_{A}\left\{1+\frac{-{m_{0}\omega }_{p}^{2}+k_{s}+d_{s}\omega_{p}j}{d_{0}\omega_{p}j+k_{0}}\right\}-{m_{0}\omega }_{p}^{2}+k_{s}+d_{s}\omega_{p}j} \end{array}$$

Consequently, the measured pulse signal using the sensor is *x*_2*C*_(*t*). As such, the distortions caused by MA at the TCS stack *x*_2*MA*_(*t*) and the wall *x*_1*MA*_(*t*) become

(9a) $$x_{2MA}\left(t\right)=x_{2TVSP}\left(t\right)+x_{2b}(t)-z_{b}(t)\;\mathrm{with}\;x_{2TVSP}\left(t\right)=x_{2M}\left(t\right)-x_{2C}\left(t\right)$$

(9b) $$x_{1MA}\left(t\right)=x_{1TVSP}\left(t\right)+x_{1b}\left(t\right)\;\mathrm{with}\;x_{1TVSP}\left(t\right)=x_{1M}\left(t\right)-x_{1C}\left(t\right)$$

where *x*_1*TVSP*_(*t*) and *x*_2*TVSP*_(*t*) denote the TVSP-generated distortion at the sensor and the wall, respectively.

As revealed in Figure 2c, in essence, there are two inputs in pulse measurement: MA *z_b_*(*t*) on the sensor substrate and the true pulse signal *F*(*t*) on the arterial wall. MA causes the length change *x*_2*b*_(*t*) − *x*_1*b*_(*t*) and, consequently, TVSP of the TCS stack. As such, MA is manifested in a measured pulse signal as BD *x*_2*b*_(*t*) − *z_b_*(*t*) and TVSP-generated *x*_2*TVSP*_(*t*), with the latter riding on each harmonic of the true pulse signal, as shown in Equation (5).

As Figure 2d illustrates, a measured pulse signal results from the intricate interaction of all the factors involved in pulse measurement: tissue, sensor, alignment, *P_c_*, MA (*z_b_*(*t*)), and the artery and its pulse signal. The collective behavior of the tissue, sensor, alignment, and *P_c_* can be quantified as the system parameters of the TCS stack: *m*_0_, *k*_0_, *d*_0_, *k_s_*, and *d_s_*. While *P_c_* sets the static length *x_dc_* of the TCS stack and can be used to tune the values of *m*_0_, *k*_0_, and *d*_0_ for a clear pulse signal (i.e., larger amplitude), MA is essentially unwanted time-varying contact pressure, causing *x*_2*b*_(*t*) − *x*_1*b*_(*t*) and *m*(*t*), *k*(*t*), and *d*(*t*), of the TCS stack. It is worth noting that this analytical model of MA is applicable for measured pulse signals using other types of sensors, such as accelerometers and PPG sensors, simply by replacing the 2DOF model in Figure 2 with its counterparts for these two types of sensors [12].

### 2.3. TFA Algorithm of a Measured Pulse Signal Using the HVD Method

As shown in Equation (5), each harmonic in the true pulse signal is modulated by TVSP, so each constant harmonic in the true pulse signal [2,16] becomes a time-varying harmonic in the measured pulse signal. Then, the TFA of a measured pulse signal becomes necessary to clarify the effect of TVSP on it. To this end, the Hilbert vibration decomposition (HVD) is chosen for such analysis due to its simplicity [20,21,24,25]. Its working principle and implementation are well documented in the literature [21,24,26] and thus is omitted here. Due to the proximity of harmonics in a pulse signal, EMD fails to effectively separate the harmonics without additional techniques [24]. The analyzed results using the CWT method are affected by the pre-defined wavelet function [19]. In contrast, the HVD method does not need any wavelet function and separate harmonics based on their amplitudes instead of frequencies.

**Figure 3:**
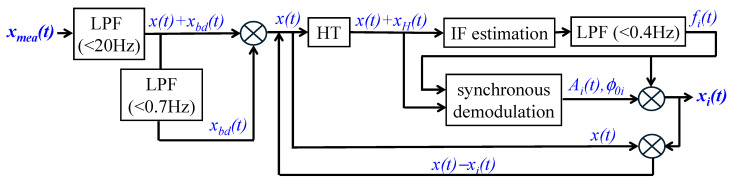
A signal-processing algorithm for the TFA of a measured pulse signal using the HVD method.

Figure 3 depicts the signal-processing algorithm for analyzing a measured pulse signal using the HVD method. A measured pulse signal *x_mea_*(*t*) contains BD *x_bd_*(*t*) (<0.7 Hz) and high-frequency noise *x_hf_*(*t*) (>20 Hz):

(10) $$x_{mea}\left(t\right)=x\left(t\right)+x_{bd}\left(t\right)+x_{hf}\left(t\right)$$

where *x*(*t*) is the portion corresponding to the true pulse signal:

(11) $$x\left(t\right)=\sum_{i=1}^{N}x_{i}\left(t\right)=\sum_{i=1}^{N}A_{i}\left(t\right)\cdot cos(2\pi \cdot \int f_{i}(t)dt+\phi_{0i})$$

where *x_i_*(*t*) denotes the *i*-th harmonic in a measured pulse signal with instant frequency *f_i_*(*t*), instant amplitude *A_i_*(*t*), and instant initial phase *ϕ*_0*i*_.

First, a measured pulse signal *x_mea_*(*t*) goes through a low-pass filter (LPF) to remove *x_hf_*(*t*). Afterward, the filtered signal goes through another LPF to remove *x_bd_*(*t*). Then, the signal *x*(*t*) in the range of (0.7 Hz, 20 Hz) serves as the input signal for TFA. A Hilbert transform (HT) is conducted on *x*(*t*) to obtain its transformed counterpart *x_H_*(*t*). Instant frequency (IF) estimation is conducted on *x(t) + x_H_*(*t*) to estimate an instant frequency, which further goes through an LPF (<0.4 Hz) to obtain *f_i_*(*t*). Synchronous demodulation is conducted on *x*(*t*), *x_H_*(*t*), and *f_i_*(*t*) to estimate *A_i_*(*t*) and *ϕ_0i_*. Using Equation (11), *x_i_*(*t*) is obtained. Afterward, *x*(*t*) − *x_i_*(*t*) serves as the new input for the HVD method to obtain *x*_*i*+1_(*t*), and this process keeps repeating until the *N*-th harmonic is obtained.

HVD decomposes a multi-component signal into separate components in the decreasing order of amplitude [20]. As to an arterial pulse signal, the amplitude of its harmonics exhibits a clear decreasing trend with the harmonic order for the first several harmonics [2]. Thus, the first component extracted will be the 1st harmonic, which will be followed by the subsequent harmonics. The effectiveness of the HVD method depends on the amplitude difference between components. Since the amplitudes of the higher harmonics in an arterial pulse signal are at similar levels, the HVD method is ineffective in separating them from each other, as will be seen in Section 3.

### 2.4. Calculation

To validate the HVD method for the TFA of a measured pulse signal, the pulse signal at the carotid artery (CA) for healthy 25 yr-old virtual subjects [2] is chosen as the true pulse signal to generate a calculated pulse signal with pre-defined MA. To account for the effect of respiration on a measured pulse signal, the frequency modulation (FM) of a pulse signal with respiration is included in this true pulse signal [19]:

(12) $$\begin{array}{c} \Delta p\left(t\right)=\sum_{i=1}^{N}A_{i}\cdot cos(2\pi {if}_{C}t+\phi_{i}+0.2\sin\left(2\pi 0.2t\right))\\ \mathrm{with}\;x_{0}\left(t\right)=\frac{a^{2}}{Eh}\Delta p\left(t\right) \end{array}$$

where the frequency of the *i*-th harmonic is *i·f_C_* − 0.04cos(2π0.2t), with *f_C_* being the frequency of the 1st harmonic and 0.2 Hz being the respiration rate (RR), and *A_i_* and *ϕ_i_* are the *i*-th constant amplitude and constant initial phase, respectively. When free of measurement, *x*_0_(*t*) is the arterial wall displacement [12], different from the one affected by the TCS stack in Figure 1b.

The nominal values for the TCS stack in [12] are used here: *k*_0_ = 1/6· *k_A_*, *r*_0_ = *f*_0_/*f_C_* = 2, and *ζ*_0_ = 1.5, where *f*_0_ and *ζ*_0_ are the nominal frequency and damping factor of the 1DOF system, respectively. The choice of these values is based on the related experimental findings [12]. Note that *z_b_*(*t*) on the sensor substrate is from a measured pulse signal (see Figure 7). Owing to the geometrical and anatomical complexity of the tissue, no mathematical relation can be expected between *x*_2*b*_(*t*) − *x*_1*b*_(*t*) and *TVSP*. Here, we assume a linear relation between them:

(13) $$\;k\left(t\right)=-\frac{k_{0}}{3}\cdot \frac{x_{2b}\left(t\right)-x_{1b}\left(t\right)}{\max\left(x_{b}\left(t\right)\right)}\;\;c\left(t\right)=-\frac{c_{0}}{3}\cdot \frac{x_{2b}\left(t\right)-x_{1b}\left(t\right)}{\max\left(x_{b}\left(t\right)\right)};\;m\left(t\right)=-\frac{m_{0}}{2}\cdot \frac{x_{2b}\left(t\right)-x_{1b}\left(t\right)}{\max\left(x_{b}\left(t\right)\right)}$$

Note that *m*(*t*) varies faster with *x*_2*b*_(*t*) − *x*_1*b*_(*t*) than *k*(*t*) for reduced natural frequency with BD [12]. Based on these parameters, a calculated pulse signal with pre-defined MA to mimic a measured pulse signal can be obtained using the analytical model.

All the calculations are conducted in MATLAB. The built-in function ODE45 is utilized for the time-domain calculations for a calculated pulse signal with pre-defined MA. Fast Fourier transform (FFT) analysis is conducted for the frequency spectrum of a measured pulse signal. The related built-in functions in MATLAB are utilized to carry out the TFA of a measured pulse signal. For the clarity of the time-frequency information of extracted signals, wavelet synchrosqueezed transform (WSST) is used. To avoid introducing unknown distortion to a measured pulse signal, no denoising or smoothing is conducted on a measured pulse signal to remove broadband noise in it. Note that synchronous demodulation in the HVD method can greatly alleviate broadband noise [26].

## 3. Results

### 3.1. Calculated Pusle Signals with Pre-Defined MA

**Figure 4:**
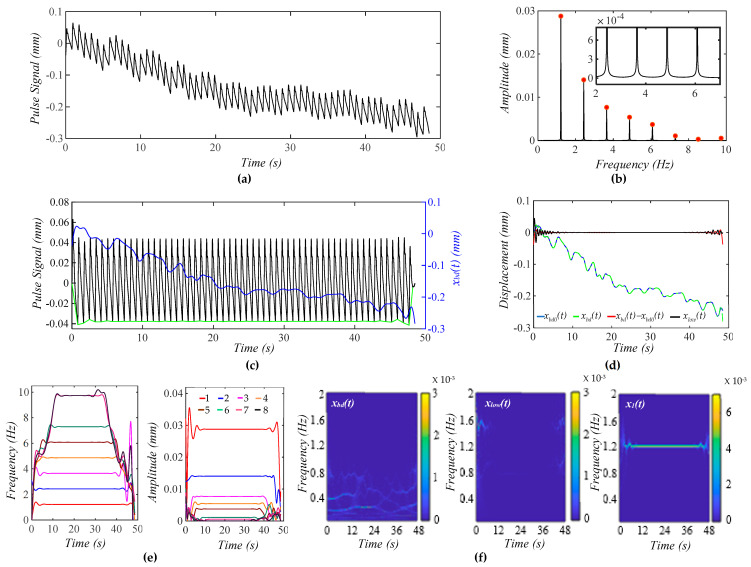
A calculated pulse signal with pre-defined BD only. (**a**) The pulse signal in time domain; (**b**) frequency spectrum of the pulse signal with no *x_bd_*(*t*); (**c**) the pulse signal with no *x_bd_*(*t*), *x_bd_*(*t*), and baseline (green) in time domain; (**d**) *x_bd_*(*t*), *x*_*bd*0_(*t*), *x_bd_*(*t*) − *x*_*bd*0_(*t*), and *x_low_*(*t*); (**e**) *f_i_*(*t*) and *A_i_*(*t*) of the pulse signal; and (**f**) WSST plot of *x_bd_*(*t*), *x_low_*(*t*), and *x*_1_(*t*).

Figure 4a shows a calculated pulse signal based on Equation (12) with pre-defined BD *x*_*bd*0_(*t*) only. The extracted BD from the pulse signal is *x_bd_*(*t*). As shown in Figure 4b, the frequency spectrum of the pulse signal with the removal of *x_bd_*(*t*) reveals all the harmonics in the pulse signal. Figure 4c shows the pulse signal with no *x_bd_*(*t*) and *x_bd_*(*t*) in the time domain. Evidently, the pulse signal with no *x_bd_*(*t*) is with a flat baseline. Figure 4d shows that the extracted *x_bd_*(*t*) is identical to *x*_*bd*0_(*t*). Note that *x_low_*(*t*) represents the signal below *f_C_* + 0.4 Hz that is left out by *x_bd_*(*t*) and *x*_1_(*t*) in the pulse signal. Clearly, *x_bd_*(*t*) and *x*_1_(*t*) capture BD and the first harmonic with good accuracy. As shown in Figure 4e, the HVD method only works for extracting instant frequencies and amplitudes for the first five harmonics. The dramatic changes at the ends of these instant variables arise from the end effects of the Hilbert transform and do not carry any physical meanings. The instant frequency and instant amplitude of each harmonic remain constant. Figure 4f shows the WSST plots of *x_bd_*(*t*), *x_low_*(*t*), and *x*_1_(*t*). Note that *x_low_*(*t*) does not carry signals at *f_C_* ± RR.

**Figure 5:**
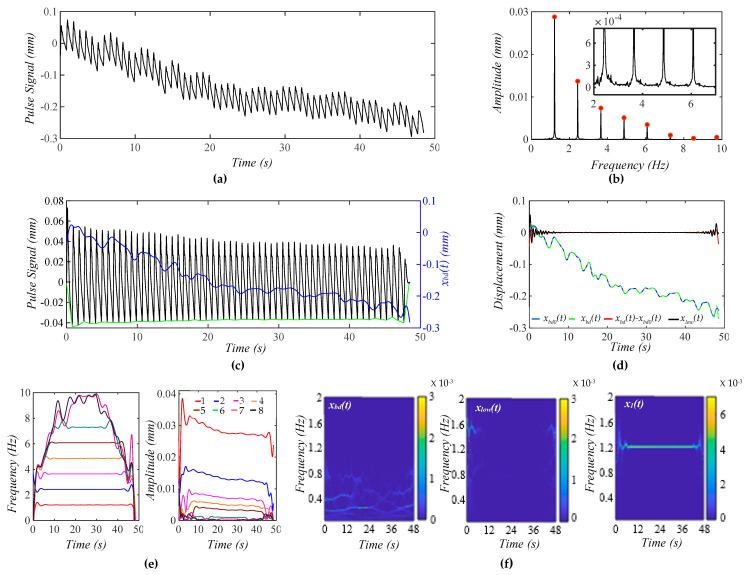
A calculated pulse signal with pre-defined MA (BD+TVSP) and no respiration. (**a**) The pulse signal in time domain; (**b**) frequency spectrum of the pulse signal with no *x_bd_*(*t*); (**c**) the pulse signal with no *x_bd_*(*t*),*x_bd_*(*t*), and baseline (green) in time domain; (**d**) *x_bd_*(*t*), *x*_*bd*0_(*t*), *x_bd_*(*t*) − *x*_*bd*0_(*t*), and *x_low_*(*t*); (**e**) *f_i_*(*t*) and *A_i_*(*t*) of the pulse signal; and (**f**) WSST plots of *x_bd_*(*t*), *x_low_*(*t*), and *x*_1_(*t*).

Figure 5a shows a calculated pulse signal with pre-defined MA (BD and TVSP). As shown in Figure 5b, there are small sidebands at the bottom of each harmonic due to TVSP. Figure 5c illustrates the pulse signal with no *x_bd_*(*t*) and *x_bd_*(*t*) in the time domain. The baseline becomes non-flat, due to harmonic-MA-coupling. The decreasing trend of *x_bd_*(*t*) indicates a decreasing *P_c_* over time. The amplitude of the pulse signal reveals a slowly decreasing trend with this decreasing *P_c_*. It must be pointed out that due to TVSP riding on each harmonic of the true pulse signal, namely harmonic-MA-coupling, the baseline in the pulse signal with no *x_bd_*(*t*) becomes non-flat.

As shown in Figure 5d, the extracted *x_bd_*(*t*) and the input *x*_*bd*0_(*t*) are very close. Given the small *x_low_*(*t*), the extracted *x*_1_(*t*) and *x_bd_*(*t*) are pretty accurate. As shown in Figure 5e, TVSP does not cause any time-varying frequency but time-varying amplitude for the first five harmonics. The instant amplitudes of these harmonics decrease over time, following the decreasing trend of *x_bd_*(*t*). The decreasing pace of the instant amplitude is the highest for the first harmonic and declines with the harmonic order. As shown in Figure 5f, *x_low_*(*t*) does not carry signals at *f_C_* ± RR.

**Figure 6:**
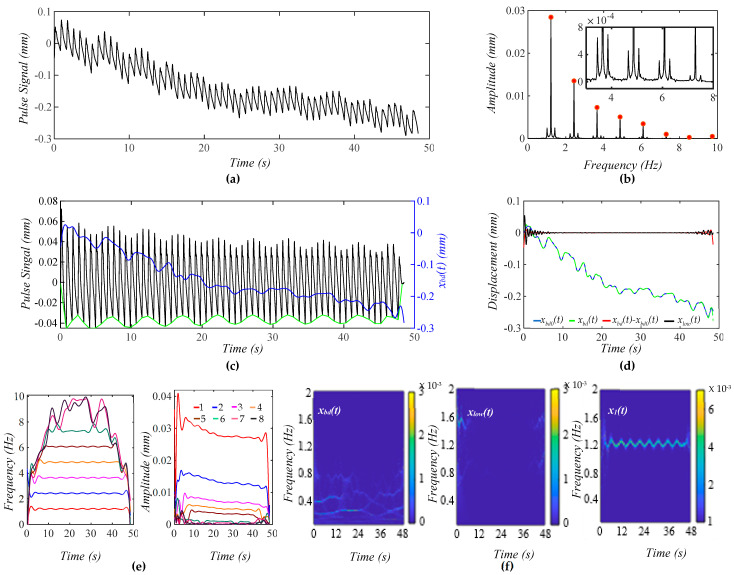
A calculated pulse signal with pre-defined MA (BD+TVSP) and respiration. (**a**) The pulse signal in time domain; (**b**) frequency spectrum of the pulse signal with no *x_bd_*(*t*); (**c**) the pulse signal with no *x_bd_*(*t*), *x_bd_*(*t*), and baseline (green) in time domain; (**d**) *x_bd_*(*t*), *x*_*bd*0_(*t*), *x_bd_*(*t*) − *x*_*bd*0_(*t*), and *x_low_*(*t*); (**e**) *f_i_*(*t*) and *A_i_*(*t*) of the pulse signal; and (**f**) WSST plots of *x_bd_*(*t*), *x_low_*(*t*), and *x*_1_(*t*).

Figure 6a shows a calculated pulse signal with MA (BD+TVSP) and respiration. As shown in Figure 6b, there are sharp sidebands around each harmonic arising from respiration, besides the small sidebands from TVSP. As shown in Figure 6c, the amplitude of the pulse signal reveals a slowly decreasing trend with this decreasing *x_bd_*(*t*). As compared with Figure 5c, the non-flatness of the baseline in the pulse signal with no *x_bd_*(*t*) stems from the harmonic-MA-coupling and respiration-modulation. As shown in Figure 6d, *x_bd_*(*t*) and *x*_*bd*0_(*t*) are very close. Meanwhile, the small *x_low_*(*t*) indicates the accuracy in the extracted *x*_1_(*t*) and *x_bd_*(*t*). As shown in Figure 6e, the instant frequency of all the harmonics reveals the same time-varying pattern, indicating that the time-varying frequency of each harmonic solely results from respiration. Comparison between Figure 5e and Figure 6e implies that TVSP solely causes the time-varying amplitude, and the effect of TVSP on the instant amplitude of each harmonic is unaffected by the presence of respiration. As shown in Figure 6f, *x_low_*(*t*) does not carry signals at *f_C_* ± RR despite the added respiration.

### 3.2. Measured Pulse Signals Pre-Exercise and 5 min Post-Exercise

**Figure 7:**
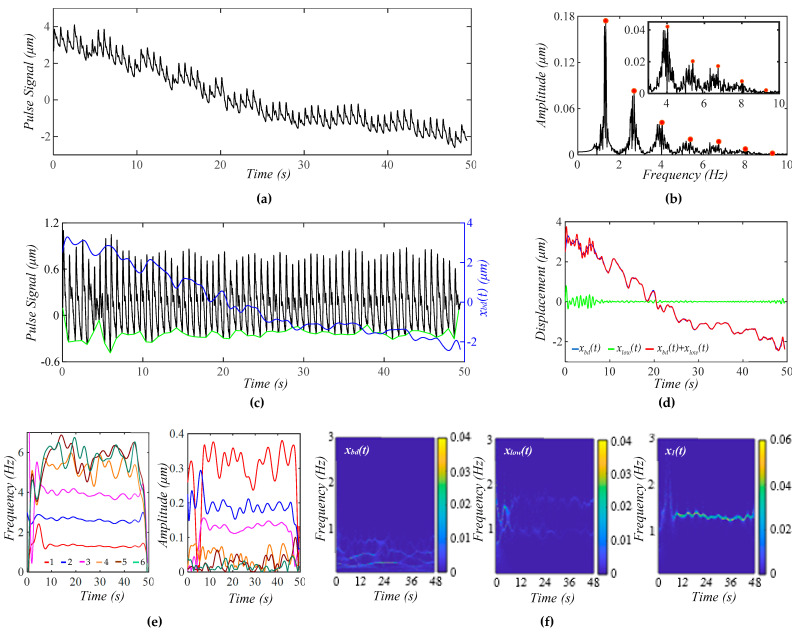
A measured pulse signal on a 32 yr-old healthy male subject pre-exercise. (**a**) The pulse signal in time domain; (**b**) frequency spectrum of the pulse signal with no *x_bd_*(*t*); (**c**) the pulse signal with no *x_bd_*(*t*), *x_bd_*(*t*), baseline (green)in time domain; (**d**) *x_bd_*(*t*), *x_low_*(*t*), and *x_bd_*(*t*) + *x_low_*(*t*); (**e**) *f_i_*(*t*) and *A_i_*(*t*) of the pulse signal; and (**f**) WSST plots of *x_bd_*(*t*), *x_low_*(*t*), and *x*_1_(*t*).

Figure 7a shows a measured pulse signal at rest on a 32yr-old healthy male subject pre-exercise. Figure 7b shows the frequency spectrum of the pulse signal with the extracted *x_bd_*(*t*) being removed, revealing uneven, large sidebands around each harmonic. As shown in Figure 7c, although *x_bd_*(*t*) shows a decreasing trend over time, the amplitude of the pulse signal with no *x_bd_*(*t*) does not show a similar decreasing trend, and the pulse signal contains a non-flat baseline. Figure 7d compares the difference between *x_bd_*(*t*) and *x_low_*(*t*), showing *x_low_*(*t*) << *x_bd_*(*t*). As shown in Figure 7e, the HVD method is effective in separating only the first three harmonics. Their instant frequencies vary with time to similar small extents, but their instant amplitudes vary with time to a much larger extent, and this time-varying extent decreases with the harmonic order. Overall, the time-varying amplitude of these harmonics remains flat over time, which is consistent with the amplitude of the pulse signal not showing any changing trend over time. Figure 7f shows the WSST plots of *x_bd_*(*t*), *x_low_*(*t*), and *x*_1_(*t*). Note that this *x_bd_*(*t*) is used as the pre-defined *x*_*bd*0_(*t*) = 5 × *x_bd_*(*t*) in Figure 4 and as *z_b_*(*t*) = 10 × *x_bd_*(*t*) in the analytical model (to generate a noticeable decreasing trend of *x_bd_*(*t*) over time) in Figure 5 and Figure 6.

As shown here, *x_bd_*(*t*) contains multiple low-frequency signals with a time-varying amplitude and time-varying frequency, which conceivably lead to TVSP, with great complexity in terms of its frequency and amplitude. Based on the analyzed results on the calculated pulse signals, the time-varying frequency of *x*_1_(*t*) should arise from respiration. This conclusion is further supported by *x_low_*(*t*), which contains two signals at ~1 Hz and ~1.6 Hz.

**Figure 8:**
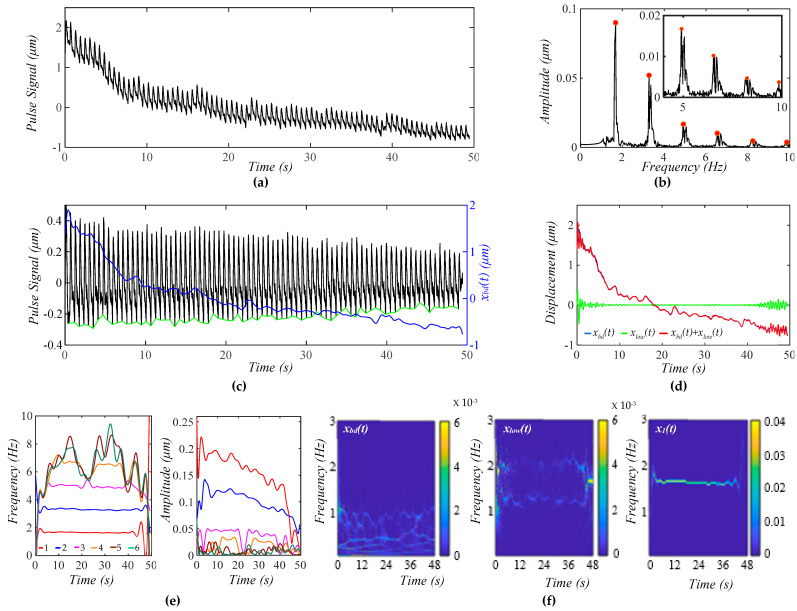
A measured pulse signal (from the 1st transducer in Figure 9) on a 32 yr-old healthy male subject 5 min post-exercise. (**a**) The pulse signal in time domain; (**b**) frequency spectrum of the pulse signal with no *x_bd_*(*t*); (**c**) the pulse signal with no *x_bd_*(*t*), *x_bd_*(*t*), and baseline (green) in time domain; (**d**) *x_bd_*(*t*), *x_low_*(*t*), and *x_bd_*(*t*) + *x_low_*(*t*); (**e**) *f_i_*(*t*) and *A_i_*(*t*) of the pulse signal; and (**f**) WSST plots of *x_bd_*(*t*), *x_low_*(*t*), and *x*_1_(*t*).

Figure 8a shows a measured pulse signal at rest on the same subject 5 min post-exercise. Figure 8b shows the frequency spectrum of the pulse signal with no *x_bd_*(*t*), revealing uneven, relatively small sidebands around each harmonic, as compared to Figure 7b, indicating that TVSP relative to this pulse signal is small, as compared to its counterpart in Figure 7. As shown in Figure 8c, the amplitude of the pulse signal with no *x_bd_*(*t*) follows the decreasing trend of *x_bd_*(*t*) over time, and the pulse signal contains a non-flat baseline. Figure 8d compares the difference between *x_bd_*(*t*) and *x_low_*(*t*) with *x_low_*(*t*) << *x_bd_*(*t*), indicating the accuracy in the extracted *x_bd_*(*t*) and *x*_1_(*t*).

As shown in Figure 8e, the HVD method is effective in separating the first four harmonics. However, a small uptick in MA at ~24 s and ~39 s mixes up the third and fourth harmonics. As to these four harmonics, their instant frequencies vary with time to similar small extents, and their instant amplitudes show a decreasing trend over time, which is consistent with the amplitude of the pulse signal going down in Figure 8c. The instant amplitudes of the four harmonics do not swing as much as their counterparts in Figure 7e. As shown in Figure 8f, *x_bd_*(*t*) contains multiple low-frequency signals with a time-varying amplitude and time-varying frequency, which is different from that in Figure 7f. The time-varying frequency of *x*_1_(*t*) should arise from respiration. This conclusion is further supported by *x_low_*(*t*), which contains two signals at ~1.4 Hz and ~2.2 Hz. 

**Figure 9:**
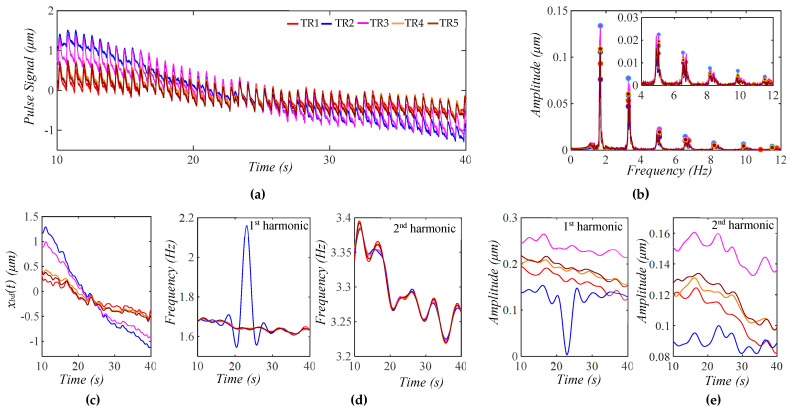
Measured pulse signals from the five transducers on a 32 yr-old healthy male subject 5 min post-exercise. (**a**) The five pulse signals in time domain; (**b**) frequency spectrum of the five pulse signals with the removal of their *x_bd_*(*t*); (**c**) *x_bd_*(*t*) in the five pulse signals; (**d**) instant frequency *f_i_*(*t*) for the 1st and 2nd harmonics; and (**e**) instant amplitude *A_i_*(*t*) for 1st and 2nd harmonics.

While Figure 8 shows the measured pulse signal from only the first transducer, Figure 9a shows the measured pulse signals from five transducers in the same measurement. As shown in Figure 9b, the amplitudes and sidebands of their harmonics vary between the signals, indicating a different TVSP between the five signals. While the peaks of the first and second harmonics from the five signals are still located at the same frequencies for these signals, the frequencies corresponding to the peaks of the third harmonic and above slightly shift between them. As shown in Figure 9c, the extracted *x_bd_*(*t*) varies between the five pulse signals. At ~24 s, the sensor experiences a sudden change. As shown in Figure 9d, except for the second transducer, the other four transducers provide almost identical instant frequencies for the first and second harmonics. However, although these four transducers show a similar decreasing trend of the instant amplitudes of the two harmonics over time, the absolute values of the instant amplitudes vary between the transducers, possibly indicating the difference in the TCS stack they sit on. The abnormal results from the second transducer are believed to come from the sudden change at ~24 s.

## 4. Discussion

### 4.1. HVD Method, BD and TVSP, and TFA

#### 4.1.1. Effectiveness of the HVD Method

As shown in Figure 4, Figure 5, and Figure 6, the HVD method is effective in separating the first five harmonics from the rest in a calculated pulse signal with BD, MA, and respiration. This is due to the amplitudes of the sixth harmonic and above being comparable. The TVSP in the two pulse signals with MA in Figure 5 and Figure 6 is quite small, causing a low-level time-varying amplitude in the first five harmonics. In contrast, the HVD method can only extract the first three harmonics from the measured pulse signal pre-exercise. The high-level time-varying amplitudes of these three harmonics indicate a large TVSP in it. This large TVSP is still small, relative to the amplitudes of these three harmonics. However, it becomes comparable to the amplitudes of the fourth harmonic and above, causing large amplitude swings and amplitude crossings between them, as shown in Figure 7e. 

As shown in Figure 8, the low-level time-varying amplitude in the first four harmonics indicates a small TVSP, which allows for the extraction of even the fourth harmonics. Yet, since the TVSP in this pulse signal is not well-below the amplitudes of the third and fourth harmonics, even a small uptick in MA at ~24 s and ~39 s mixes up the two harmonics. As such, the level of time-varying amplitude relative to the amplitudes of harmonics poses another limit on the effectiveness of the HVD method on separating harmonics in a measured pulse signal.

#### 4.1.2. BD and TVSP

In this study, an important hypothesis on MA is that MA causes not only BD but also TVSP. This hypothesis is built on the well-established theory that pre-stress alters the mechanical properties of a solid [17]. Overlying tissue is pre-stressed by *P_c_*. MA causes time-varying variations in *P_c_* and thus a time-varying pre-stress level. Then, the mechanical properties of overlying tissue are altered by MA, leading to TVSP in the TCS stack.

The analytical model built upon this hypothesis reveals that TVSP is multiplied by each harmonic of the true pulse signal, causing uneven sidebands around each harmonic in a measured pulse signal. However, this model cannot reveal how TVSP alters the frequency and amplitude of each harmonic in a measured pulse signal. To this end, the TFA of the calculated pulse signals with pre-defined MA is conducted, showing that TVSP only causes time-varying amplitudes in each harmonic but has no effect on its frequency. Since this time-varying amplitude varies positively with the amplitude of a harmonic, it decreases with the harmonic order. Due to TVSP, a measured pulse signal with no BD should possess non-flat harmonic-MA-coupled baseline, as shown in Figure 5, Figure 7, and Figure 8. These theoretical observations are consistent with the analyzed results on the measured pulse signals. As such, the key findings on the effect of MA at rest on a measured pulse signal are as follows:

(1) TVSP causes the time-varying amplitude but has no effect on the frequency of each harmonic in a measured pulse signal;
(2) The time-varying amplitude caused by TVSP decreases with the harmonic-order;
(3) A measured pulse signal with no BD possesses a non-flat harmonic-MA-coupled baseline.

Although this study cannot extract higher harmonics from a measured pulse signal, the above findings are applicable to them, based on the analytical model.

As shown in Figure 7 and Figure 8, BD in a measured pulse signal is a combination of multiple signals with their own time-varying frequency and time-varying amplitude. Yet, despite the complexity of BD, its low frequency and additive nature to the true pulse signal make it much easier to estimate with good accuracy. Instead, it is TVSP that is difficult to estimate. The comparison between Figure 5, Figure 6, and Figure 7 shows that the assumed values for *m*_0_, *k*_0_, and *d*_0_ and *m*(*t*), *k*(*t*), and *d*(*t*) of the TCS stack are far off from the reality. As shown in their frequency spectrum, the measured pulse signals contain broadband noise and may add some errors to the analyzed results.

#### 4.1.3. TFA Versus Time-Domain and Frequency-Domain Analysis

As shown in Sec. 3, the amplitude and waveform of a measured pulse signal are greatly distorted by MA. A measured pulse signal with BD removal still shows noticeable variations between pulse cycles. These variations attest to the great sensitivity of a measured pulse signal to MA in the time domain [3,9,15]. As shown in Figure 9, the frequency-domain information of a measured pulse signal is relatively resilient to MA, in the sense that the extracted peaks of the first and second harmonics in a measured pulse signal are still located at the corresponding frequencies in the true pulse signal, unaffected by the variations in the TCS stack and TVSP. Meanwhile, the frequencies of the extracted peaks of the higher harmonics differ only slightly between the signals. However, the value of each peak is smeared by its accompanying sidebands and is thus not indicative of the amplitude of the corresponding harmonic in the true pulse signal, based on Equation (8b). In contrast, the TFA of a measured pulse signal allows for the extraction of much more accurate information on the frequency and amplitude of each harmonic in a measured pulse signal. Only the TFA makes clear the effect of TVSP on the frequency and amplitude of each harmonic in a measured pulse signal.

### 4.2. Implications for Measured Pulse Signals and Clinical Applications

As shown in Figure 2d, a measured pulse signal results from the intricate interaction of all the factors involved in pulse measurement. All these factors contribute to the TCS stack and can be quantified into a 1DOF system. When free of MA, the TCS stack serves as a harmonic-dependent transfer function, as shown in Equation (8b), providing a theoretical basis for measurement variations between individuals, the sensor used, alignment, as well as *P_c_* [5,12]. In other words, a measured pulse signal always deviates from the true pulse signal in an artery due to the TCS stack. MA adds another layer of complexity to this harmonic-dependent transfer function by contributing TVSP to the TCS stack. Reducing *P_c_* may alleviate such deviation but risks acquiring a measured pulse signal with severe noise and high-level MA [12].

The effect of the TCS stack on a measured pulse signal is twofold [12]: (1) affecting the true pulse signal, and (2) affecting the relationship between the affected true pulse signal and the measured pulse signal. The first effect is shown in the experimental finding: excessive *P_c_* (the effect of *P_c_* is fully accounted for by the values of *m*_0_, *k*_0_, and *d*_0_ of the TCS stack) suppresses the true pulse signal and leads to a measured pulse signal with a smaller amplitude [3,5]. The second effect is shown in the experimental finding [5]: it is difficult to acquire a clear pulse signal over thicker overlying tissue (a large stiffness). The second effect of the TCS stack originates from the deformability of the TCS stack in pulse measurement. Accounting for the deformability of the TCS stack leads to quantifying TVSP as another contribution of MA to the TCS stack and provides a theoretical basis for the uneven sidebands around each harmonic and the time-varying amplitude of each harmonic in a measured pulse signal.

The effect of *P_c_* on a measured pulse signal is intricate. It is well-established that, as *P_c_* increases from low to high, the measured pulse amplitude goes up until *P_c_* reaches a certain value (optimal *P_c_*), where the measured pulse amplitude is at maximum and will decrease when *P_c_* is beyond this value [3,5]. As shown in Figure 7c, BD decreasing indicates that *P_c_* is decreasing over time. Since the measured pulse amplitude remains unchanged, it may imply that *P_c_*, at the beginning of the measurement, suppressed the true pulse signal in the artery to some extent. However, this was not known prior to the examination of the measured pulse signal. In practice, *P_c_* cannot be controlled as well as those experimental studies focused on the effect of *P_c_* [5]. As shown in Figure 8c, *P_c_* also decreases over time, and the measured pulse amplitude decreases over time, too. The related CV physiology [11] shows that the pulse amplitude decreases over time post-exercise. This pulse signal was measured at 5 min post-exercise. The decreasing trend of the pulse amplitude over time is believed to at least partially be attributed to exercise, but whether *P_c_* contributes to this trend is unclear.

As shown in Section 2, there co-exist six unknowns in the TCS stack: three nominal parameters: *k*_0_, *d*_0_, and *m*_0_, and three TVSPs: *k*(*t*), *d*(*t*), and *m*(*t*). There are no direct theoretical relations between them. The complexity in the relations between them in Equation (5) shows that the sensitivity of a measured pulse signal to one unknown will be greatly altered by the values of the other five unknowns. Thus, extraction of the values for the six unknowns needs to be conducted together from a measured pulse signal. Such extraction for multiple unknowns from a measured pulse signal amounts to a significant challenge in the field of TFA for the system identification of dynamic/structural systems [18], where output-only signal-processing algorithms are intensively pursued for different applications. This explains the reason why the values of *k*_0_, *d*_0_, and *m*_0_ used here are assumed based on experimental findings [12], and the values of *k*(*t*), *d*(*t*), and *m*(*t*) are assumed based on BD. To make things worse, the interaction between an artery and the TCS stack above it will alter the true pulse signal *Δp*(*t*) in Figure 1 [12]. As such, further studies are needed to quantify the nominal parameters and TVSP of the TCS stack and the artery–TCS–stack interaction so that the unaffected true pulse signal can be extracted from a measured pulse signal, prior to the applications of pulse measurement as clinical routine and at-home-use for CV monitoring.

**Figure 10:**
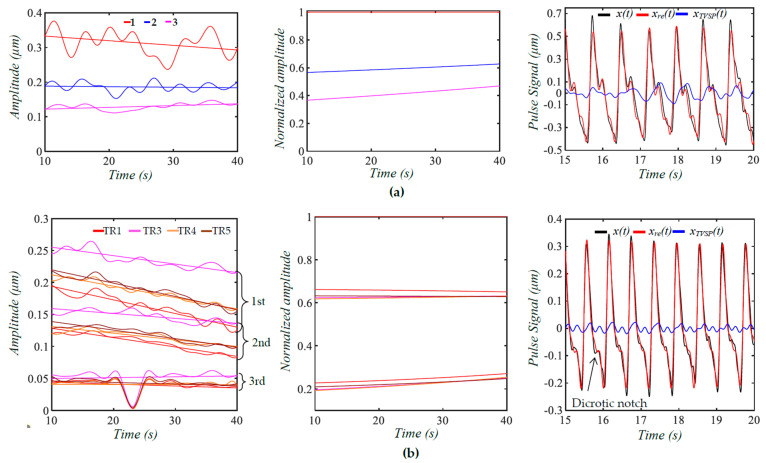
Regression lines for *A_i_*(*t*), normalized amplitude *A_i_*(*t*)/*A*_1_(*t*), and the reconstructed pulse signal from the extracted three harmonics with their regression lines as amplitude *x*(*t*): measured pulse signal with BD removal; *x_re_*(*t*): reconstructed pulse signal; and *x_TVSP_*(*t*): MA associated with the three harmonics based on time-varying amplitude. (**a**) Measured pulse signal pre−exercise; (**b**) measured pulse signals 5 minpost−exercise from four transducers (the 2nd transducer is excluded).

To date, arterial indices are mostly derived from the features of a measured pulse signal and its time derivatives in the time domain [2,3,4,5,6]. To compensate for the variations between pulse cycles, the average values of arterial indices derived from several pulse cycles are used as the measured results [3,5]. Based on this study, a regression line for the time-varying amplitude is used to represent the amplitude of each harmonic free of TVSP, as shown in Figure 10. We further reconstruct the two measured pulse signals from their three extracted harmonics with their regression lines as the amplitude. The difference between pulse cycles in the reconstructed pulse signal mainly arises from time-varying frequency of the harmonics, and MA associated with the three harmonics is lower in the measured pulse signal 5 min post-exercise, as compared to pre-exercise. Evidently, these three low harmonics capture the lowered dicrotic notch 5 min post-exercise, as compared to pre-exercise, which is well-documented in the related clinical studies [11]. However, some fine features in the measured pulse signals are missing in the reconstructed signals, implying the need to extract higher harmonics.

Given the dependence of the pulse amplitude on the TCS stack, the amplitude of higher harmonics is normalized to the amplitude of the first harmonic for discerning CV conditions, as shown in Table 1. Compared to pre-exercise, the second harmonic amplitude is slightly increased (~0.6 vs. ~0.63), but the third harmonic amplitude is greatly reduced (~0.41 vs. ~0.22) 5 min post-exercise. Thus, the change in dicrotic notch in Figure 10 may be attributed to the changes in the amplitudes of the three harmonics. Such a normalized amplitude difference is robust to the variation between the four transducers. Due to TVSP, the peaks of the harmonics from the FFT analysis are all lower than their counterparts from TFA. It must be noted that any variation in the values of *k*_0_, *d*_0_, and *m*_0_ (i.e., tissue, sensor, and alignment) will alter these normalized amplitudes to some extent. Moreover, the interaction between the artery and the TCS stack will affect the true pulse signal in the artery to some extent [12]. In the future, TFA needs to be conducted on the measured pulse signals of more subjects to evaluate these new arterial indices with statistical significance.

### 4.3. Comparison with the Related Studies in the Literature

**Figure 11:**
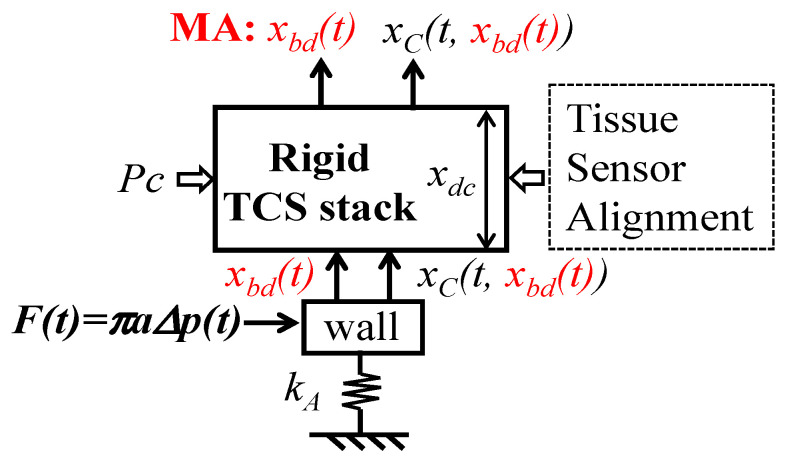
Current consensus on the effect of MA on a measured pulse signal.

To the best knowledge of the authors, in all the existing studies on MA in a measured pulse signal [3,13,14,15], regardless of the measurement instruments used, including tonometry, ultrasound, and PPG sensors, MA is equated solely with BD. Figure 11 depicts the current consensus on the effect of MA on a measured pulse signal, where the TCS stack is considered rigid and accounts for the *P_c_*, tissue, sensor, and alignment. Upon *P_c_*, the TCS stack of length *x_dc_* is established and transmits the true pulse signal from the artery to the sensor at the top of the TCS stack. When free of MA, the pulse signal *x_C_*(*t*) in the artery is identical to the one detected by the sensor, and measurement variations are solely due to the effect of the TCS stack on the true pulse signal. This is contradictory to the experimental finding that thicker overlying tissue leads to a smaller measured amplitude.

When MA appears, it presents as the displacement *x_bd_*(*t*) of the TCS stack and shifts the TCS stack relative to the artery, and thus, the true pulse signal is also affected by *x_bd_*(*t*):*x_C_*(*t*, (*x_bd_*(*t*)). As such, a measured pulse signal is *x_bd_*(*t*) + *x_C_*(*t, x_bd_*(*t*)). Such account of *x_bd_*(*t*) in the measured pulse signal leads to two conclusions: (1) A measured pulse signal with no *x_bd_*(*t*) (i.e., *x_C_*(*t, x_bd_*(*t*)) possesses a flat baseline; (2) variations between pulse cycles stem from the effect of *x_bd_*(*t*) on the true pulse signal.

Based on these two conclusions, various signal-processing algorithms are developed to filter *x_bd_*(*t*) (<0.7 Hz) out of a measured pulse signal (>1 Hz) [14,15]. Usually, the filtered pulse signal contains a non-flat baseline, which is thought to arise from the errors in estimated *x_bd_*(*t*), due to proximity of *x_bd_*(*t*) to the first harmonic in the pulse signal. To correct this non-flat baseline, a polynomial curve is estimated based on the start/end of the pulse cycle and is subtracted from the filtered pulse signal to obtain a pulse signal with a flat baseline [3,14,15]. Certainly, this polynomial curve is a low-frequency signal and adds distortion to the filtered pulse signal. As can be seen here, the filtered pulse signal with a non-flat baseline is not due to errors in the estimated *x_bd_*(*t*). Instead, it is TVSP that causes this non-flat baseline. Currently, the variations between pulse cycles from the effect of *x_bd_*(*t*) on the true pulse signal are tackled by using the average from several pulse cycles in *x_C_*(*t, x_bd_*(*t*)) to represent the measured results. However, this treatment ignores the effect of *x_bd_*(*t*) on the TCS stack and then its effect on the harmonic-dependent transfer function in Equation (8b).

Since estimated *x_bd_*(*t*) from various signal-processing algorithms is believed to be inaccurate, another solution is to add a reference sensor next to the sensor for pulse measurement to record MA [9,13]. An accelerometer usually fails to capture MA at rest with desired accuracy [9]. A reference PPG sensor is used to capture MA in pulse measurement using a PPG sensor [9]. As shown in Figure 9, although there is interference between the five transducers, due to their integration into one body, it is reasonable to believe that the difference in *x_bd_*(*t*) and TVSP between the measured signals from the five transducers, at least partially, stem from the fact that they sit on different TCS stacks and their alignments with the artery are also different. Then, the same MA causes different responses to the five transducers. As such, it is believed that MA varies between the reference PPG sensor and the PPG sensor for pulse measurement.

### 4.4. Study Limitations

There are six major study limitations in this study. First, linear behavior of the TCS stack is assumed. Second, all the values of the nominal parameters of the TCS stack and the relations between BD and TVSP are assumed. The difference between the calculated pulse signals with pre-defined MA and the measured pulse signals clearly reveal that the assumed values and the assumed relations greatly differ from their actual values. Nonetheless, the analyzed results of the measured pulse signals qualitatively validate the existence of TVSP and reveal its effect on the harmonics in the signals, and the analytical model offers an analytical framework for identifying the actual values of the TCS stack and TVSP in the future via system identification techniques. If needed for a better fit, the nonlinear behavior of the TCS stack can be incorporated into the model.

Third, the associated electronic noise and transduction in the sensor are neglected. Fourth, the high harmonics cannot be analyzed by the HVD method. As compared to the low harmonics, the signal-to-noise ratio in these high harmonics is much lower and may greatly undermine the information of TVSP in them. Nevertheless, as shown in Figure 10, high harmonics carry the fine features in a measured pulse signal and need to be analyzed for their clinical and physiological implications. In the future, further studies on the sensor noise and other methods on TFA are needed to improve the accuracy in the extracted harmonics and allow the extraction of higher harmonics in a measured pulse signal for a comprehensive picture of the effect of MA on a measured pulse signal.

Fifth, only MA at rest is considered. Currently, monitoring the heart rate and respiration rate during activities has gained great research interest [9,13]. MA during activities falls into the frequency range of an arterial pulse signal [9,13], which dramatically distorts the measured APW, heart rate, and respiration rate. Measured pulse signals during activities are usually not targeted for APW but solely for the heart rate and respiration rate. This is understandable, since MA during activities may greatly increase the level of MA and, consequently, large TVSP swings, which will greatly distort the measured APW. Furthermore, these large TVSP swings may coincide with the frequency of different harmonics in a pulse signal at different times, dramatically exacerbating the complicity of the effect of TVSP on a measured pulse signal. Finally, MA during activities may also cause intermittent tissue–sensor contact, which introduces great nonlinearity in a measured pulse signal.

Lastly, the artery–TCS–stack interaction is not considered. In fact, the TCS stack exerts force on an artery during measurement and thus alters the true pulse signal in the artery at the measurement site to some extent [12]. Such alteration varies with the artery (e.g., a stiff artery leads to less alteration), given the same TCS stack. Taken together, the ultimate challenge for the clinical application of pulse measurement is how to quantify the nominal parameters and TVSP of the TCS stack and the artery–TCS–stack interaction, if not fully, at least to some extent, so that the unaffected true pulse signal in an artery can be retrieved from a measured pulse signal.

## 5. Conclusions

In this study, by taking full account of the dynamic behavior of the transmission path (the TCS stack), MA in a measured pulse signal is quantified as the BD and TVSP of the TCS stack. An analytical model of MA in a measured pulse signal is developed to mathematically relate MA to a measured pulse signal in the time domain. A signal-processing algorithm based on the HVD method is developed for the TFA of a measured pulse signal. The algorithm is validated using a calculated pulse signal with pre-defined MA. The TFA of measured pulse signals using a microfluidic-based tactile sensor proves the existence of TVSP and provides a clearer picture of the effect of TVSP on a measured pulse signal: time-varying amplitude of each harmonic and non-flat harmonic-MA-coupled baseline. Great variations in the information of each harmonic in the time domain due to TVSP necessitates the TFA of a measured pulse signal. This study reveals that, prior to the applications of pulse measurement as clinical routine or for at-home-use, further studies on quantifying the TCS stack and TVSP and the artery–TCS–stack interaction are needed to retrieve the unaffected true pulse signal in an artery from a measured pulse signal.

## Figures and Tables

**Table 1:** Comparison of the normalized amplitude difference between FFT and TFA pre−exercise and 5 min post-exercise.

Harmonics	Pre-Exercise		5 min Post-Exercise							
			TR1		TR3		TR4		TR5	
	FFT	TFA	FFT	TFA	FFT	TFA	FFT	TFA	FFT	TFA
1st	1	1	1	1	1	1	1	1	1	1
2nd	0.506	0.595	0.322	0.657	0.619	0.627	0.578	0.623	0.561	0.630
3rd	0.244	0.415	0.196	0.238	0.186	0.220	0.168	0.218	0.165	0.225

The amplitude of the 1st, 2nd and 3rd harmonics is normalized to that of the 1st harmonic.

## Data Availability

The data that support the findings of this study are available upon reasonable request from the corresponding author. The data are not publicly available due to privacy or ethical restrictions.

## Abbreviations

The following abbreviations are used in this manuscript:

BD	Baseline drift
DOF	Degree-of-freedom
TVSP	Time-varying system parameters
MA	Motion artifacts
FFT	Fast Fourier transform
HVD	Hilbert vibration transform
TFA	Time-frequency analysis
